# Friction Performance Improvement of Phenolic/Rockwool Fibre Composites: Influence of Fibre Morphology and Distribution

**DOI:** 10.3390/ma15155381

**Published:** 2022-08-04

**Authors:** Fatma Makni, Anne-Lise Cristol, Yannick Desplanques, Riadh Elleuch

**Affiliations:** 1 Laboratory of Electro-Mechanical Systems (LASEM), National School of Engineers of Sfax (ENIS), University of Sfax, Sfax 3029, Tunisia; 2 UMR 9013-LaMcube-Laboratoire de Mécanique Multiphysique Multiéchelle, University Lille, CNRS, Centrale Lille, F-59000 Lille, France

**Keywords:** fibre morphology, microstructure heterogeneity, friction behaviour, wear mechanisms, organic composite friction materials

## Abstract

The size and morphology of reinforcing fibres have a great influence on organic brake friction composite material properties and performance. This research aims to establish the link between friction material microstructure heterogeneity induced by rockwool fibre morphology and distribution and the resulting tribological behaviour. The adopted approach is based on simplified formulations designed to limit synergistic effects by reducing the number and size distribution of constituents. Two simplified materials are developed with different rockwool fibre size and morphology. The first material is elaborated with calibrated fibre balls, and the second one is performed with separated fibres. Friction and wear behaviour are correlated with thermal phenomena in order to reveal wear mechanisms and thus understand the link between microstructural characteristics and the resulting tribological behaviour. It was found that a regular size and distribution of rockwool fibre balls induce better tribological behaviour and enhance wear resistance. Indeed, a homogeneously distributed porosity, which is induced by fibre balls, favours the development and preservation of the load-bearing plateaus in the contact. This, consequently guarantees a stable friction and a reduced wear rate. Consequently, reducing microstructural heterogeneity, resulting from rockwool fibre morphology and distribution, improves the performance of composite friction material.

## 1. Introduction

Friction materials used for brake linings are composed of numerous constituents with important diversity in terms of chemical composition, morphology, and size distribution. The performance of brake materials depends strongly on their formulation, microstructure, and properties [1,2,3]. Several studies investigated the optimisation of the friction material manufacturing process [4,5,6,7], the impact of types [8,9] and the relative amounts of raw material constituents [10,11] for the purpose of better brake friction performance. Brake pads contain reinforcing fibres to maintain strength, thermal stability, and friction properties [12,13]. In addition to fibres, binders, lubricants, fillers, and abrasive particles are included to enhance these properties. Among all ingredients, reinforcing fibres play a crucial role in determining friction material microstructure and performance [14,15]. Indeed, fibres in brake linings determine wear stability and friction optimisation under a set of dynamic operating variables, such as sliding speed and braking duration and temperature [14]. 

In addition, the morphology and arrangement of fibres present a major factor controlling composite material heterogeneity and influencing their mechanical behaviour [16,17]. Previous studies showed that the shape and size of fibres have a great impact on composite material properties. In fact, Horovistiz et al. [18] reported that the reinforcement percentage and orientation of fibres with respect to the sliding surface have an influence on the mechanical and tribological properties, such as elastic limit and stiffness.

In addition, Maciel et al. [19] demonstrated that aligned fibres enhance mechanical properties contrary to randomly oriented ones. Wu et al. [13] showed that the addition of helical sisal fibres can improve the bonding quality of the fibre–matrix interface, decrease the amount of fibre debonding and pullout, and consequently reduce the friction wear rate. In this same issue, Sharma and Bijwe [20] demonstrated that fibre arrangement and adhesion with the matrix play a significant role in the mechanical properties (stiffness, strength, failure strain, and energy storage capacity) and tribological behaviour for several fibre reinforced composites.

However, the link between microstructural heterogeneity induced by fibre morphology and arrangements and the resulting performance of composite friction material is still not well understood. Among all composite friction material constituents, rockwool fibres present an influential ingredient with friction material properties and performance, involving a great source of heterogeneity [3,16]. Indeed, they define the composite microstructural characteristics due to their multiple morphologies (fibres, shots, and balls of fibres) [16] and affect the composite cohesion because of their reinforcing role. It should be noted that during the elaboration process of composite friction materials, the size and shape of rockwool fibre bundles evolve, which induce different distributions in the material volume [21]. Consequently, the resulting material microstructure, properties, and performance are influenced by this constituent morphology and distribution. This paper focuses on the link between rockwool fibre arrangement and friction and wear behaviour of friction materials in order to improve this material’s performance.

## 2. Materials and Methods

### 2.1. Investigated Materials

**Figure 1:**
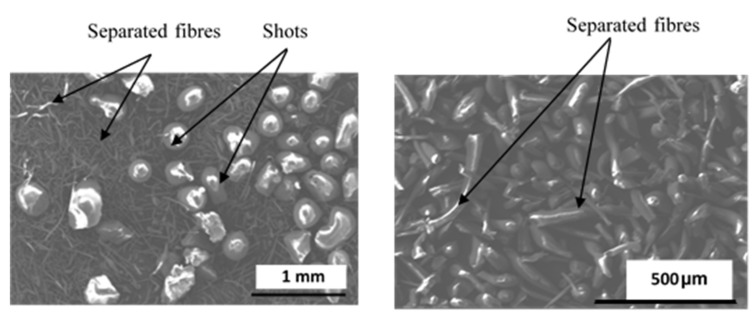
Morphologies of rockwool fibres (SEM-SE).

In order to facilitate the identification of each component of the microstructure, a simplified formulation was developed by reducing constituent number and size distribution while preserving their efficiency in braking situations [7,15]. This approach, adopted in previous studies, limits heterogeneities and facilitates the comprehension of relations between microstructure and material properties and tribological behaviour [15,16]. Six ingredients were retained (phenolic resin (HEXION, Solbiate Olona, Italy), rubber, graphite (James Durrans group, Scheffield, UK), calcium carbonate (SUPCAL 2 Tunisia), alumina (ALMATIS, Frankfurt, Germany), and rockwool fibres (Lapinus, Limburg Netherlands) as indicated in Table 1. The size distribution of constituents was reduced using the sieving method. Selected sizes of the simplified material ingredients are given in Table 1. Resin, calcium carbonate, and alumina have very fine particles (average diameter < 20 µm) and form the matrix of this composite. The manufacturing process of brake pads involves several steps (mixing, cold preforming, hot moulding, and post curing). The ingredient addition order (introduction sequence order) and mixing time were set according to the results discussed in the previous work [3,4]. It is important to note that rockwool (provided by Lapinus) comes in different morphologies; coarse particles called “shot” have a round shape, while fine fibres ranging from 5 mm to 10 mm in diameter vary over a wide range of lengths. Rockwool fibres can be found either as separated fibres or in the form of entangled ones (balls of fibres) (Figure 1). 

For this study, rockwool fibres were calibrated by the means of sieves presenting three mesh opening sizes: 400 µm, 600 µm, and 1000 µm. Two materials were examined depending on different fibre morphologies and sizes. The first one called Friction Material with Separated Fibres (FMFS) contained separated fibres with a size below 400 µm, and the second one named Friction Material with Fibre Balls (FMFBs) was made with fibres as balls in the range of 600–1000 µm (Table 1). 

All constituents, except rockwool fibres, of the two simplified materials were mixed with the same defined introduction sequences and mixing duration [3]. Mixing step was carried out using a laboratory mixer that reproduces the same mixing mechanisms of the industrial one. Rockwool fibres were incorporated in the final sequence with two different mixing durations for the investigated formulations. As reported by Makni et al. [3], to perform the mixtures of FMFS and FMFB materials, the same order and duration of introduction sequences of all constituents except rockwool fibres were applied. For FMFBs, a reduced mixing duration (48 s) of fibre balls was performed in order to preserve the initial shape and size of fibres. However, for FMFS, a longer time (460 s) of mixing was applied to improve fibre dispersion in the mixture.

**Figure 2:**
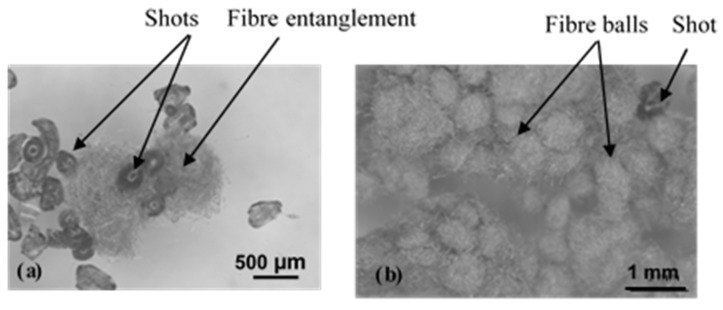
Arrangements of fibres for (**a**) FMFBs and (**b**) FMFS.

For FMFS, isolated fibres were re-entangled after their manipulation. The resulting morphologies of fibre bundles included in FMFS and FMFBs are described in Figure 2. The re-entangled fibres (Figure 2a) have a highly variable and flattened morphology compared to the regular and bulky morphology of the fibre balls. Their sizes are larger than those of fibre balls, reaching up to 2 mm. 

After mixing, a succession of steps was carried out: a cold preforming followed by a hot moulding (140 °C, 200 bars, 11 min) and finally a post-curing (160 °C, 8 h). At the end of the manufacturing process, several finishing operations were performed to obtain a flat plate 400 × 400 mm width and 16 mm thick. 

### 2.2. Microstructure Characterisation

Microstructural characterisation was performed using Scanning Electron Microscopy (SEM) using an accelerating voltage of 15 kV and a working distance of 35 mm. Samples were polished with respect to standard procedures. The purpose of this analysis is to evaluate the constituent distribution and arrangements for the studied materials.

### 2.3. Evaluation of Tribological Behaviour

#### 2.3.1. Tribometer Description

**Figure 3:**
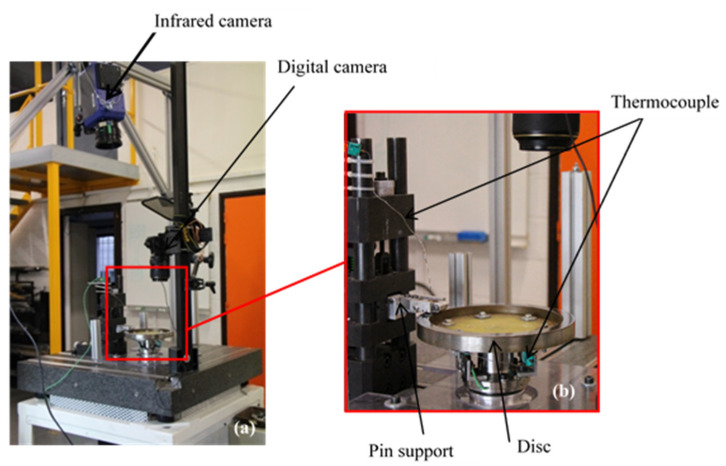
(**a**) Experimental set-up and (**b**) Pin-on-disc tribometer.

The evaluation of tribological behaviour was carried out by means of tribological tests, using pin-on-disc tribometer with a plane contact (Figure 3). This device is composed of a spindle, at the end of which the disc is mounted, driven in rotation by a “brushless” electric motor that guarantees a high degree of speed regularity. The tribometer is equipped with a device for applying the load and measuring the forces. This device comprises a 16 mm diameter clamp in which the friction material sample is fixed. The load was applied by an elastic system of 50 N/mm stiffness involving a helical spring compression. K-type thermocouples were used to measure mass temperatures in the pin and disc at 2 mm from the friction surface over the mean friction radius. An infrared camera was employed (JADE camera from CEDIP) to detect thermal localisations and correlate them with the friction evolutions. The field of observation of the infrared camera covers the friction track of the disc at the contact exit. Images of pin surface before and after friction tests were taken with a digital camera in order to obtain more information at the macroscopic scale.

#### 2.3.2. Experimental Protocol

The friction tests were performed using a pin made of the studied material rubbing against a grey cast iron disc (Figure 2). The pins, sampled from the simplified material plates, are cylinders of 16 mm in diameter and 16 mm in height. Pin axis is normal to the plate plane. The disc is made of lamellar graphite cast iron (ENGJL 250) with an average radius of friction of 100 mm and disc thickness of 20 mm. Sample preparation consists of grinding the surface of the pin and polishing the surface of the disc to grade 500 and then 1200, at 200 rpm. A control of the out-of-plane runout of the disc friction track was performed so that it is limited to about ten micrometres. 

The normal force applied to the pin is 100 N, i.e., an apparent pressure of 0.5 MPa, and the rotation speed of the disc is 600 rpm, i.e., a sliding speed of 6.3 m/s at the mean radius. A run-in phase, consisting of four 5 min sequences, precedes the tests to ensure a conforming planar contact between the disc and the pin. Disc and pin temperatures were determined using K-type thermocouples positioned on the mean friction radius, 2 mm and 3 mm below the friction surface, respectively [15].

In order to correlate the friction evolution with the tribological mechanisms and phenomena induced during the tests, friction experiments were interrupted at different durations while taking care not to exceed 100 °C for the disc temperature. The retained duration was 5 min.

#### 2.3.3. Thermal Phenomena Investigation

Thermal phenomena revealed by infrared (IR) thermography on the disc track were investigated and correlated with the friction coefficient evolution. Thermal measurements were carried out using JADE infrared camera from CEDIP, which was placed 1 m from the friction track. The IR camera field of view covered the lower right-hand portion of the disc. It does not determinate a true surface temperature because of the changes in the surface emissivity induced by wear, oxidation, and movement of the third body. However, luminance variation provides relevant information about thermal phenomena of load-bearing localisations affecting rubbed surfaces. Measurements are given in Digital Level (degrees of luminance) (DL), which corresponds to the intensity of the surface infrared radiation without being converted to surface true temperature as the surface emissivity was unknown and varies during braking [22].

The view field of the IR camera covered only the lower right-hand portion. In the resulting thermograms, grey shades from black to pink correspond to thermal radiation IR from the lowest to the highest. The thermal radiation temporal evolution was analysed by thermogram treatment on three zones: the outer radius (OR), average radius (AR), and inner radius (IR) of the disc track [23]. For each radius, we considered for the presented thermograms the thermal radiation maximum intensity emitted by an arc.

Thermogram analysis identifies load-bearing localisations (zones) and open contact on the rubbed surface. Theses localisations are detected by infrared radiation variation induced by different transitory and local phenomena affecting temperature (thermal localisation) or emissivity (wear, stripe, and third-body circulation) [24].

## 3. Results and Discussion

### 3.1. Microstructures

**Figure 4:**
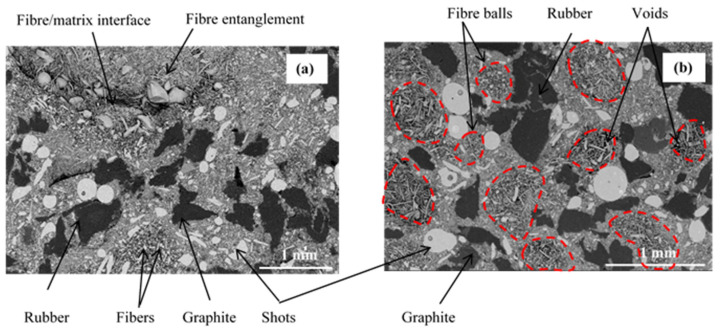
Microstructure of simplified materials (**a**) FMFS and (**b**) FMFBs.

The micrographs in Figure 4 illustrate the different constituents of the two studied materials: the fine particles (resin, calcium carbonate, and alumina) form the matrix that coats the other constituents. A surface microstructural analysis shows that the shape of fibre balls appears more regular than that observed for FMFS (Figure 4). Their outline is easily discernible, whereas it seems imperceptible for FMFS (Figure 4a). The morphology of fibre balls of FMFBs can be distinguished from that of the FMFS entanglements by the presence of voids between the fibre clusters (Figure 4b). Their distribution appears more uniform and homogeneous on the surface compared to the distribution of fibre entanglements on the surface of FMFS (Figure 4b). The majority of the fibres are dispersed in the material FMFS, but some appear entangled (Figure 4a). The separated fibres show good adhesion with the matrix, since they are for the most part embedded in the matrix. However, large fibre entanglements appear partially embedded in the matrix. This indicates that the resin particles have not penetrated to the densely entangled fibres and are therefore not fully embedded in the resin. In addition, according to previous research [16] carried out on the same materials, the microstructure of FMFBs presents a more homogeneous microstructure with regular shape and distribution of fibre balls in the material compared to FMFS.

### 3.2. Friction Behaviour

**Figure 5:**
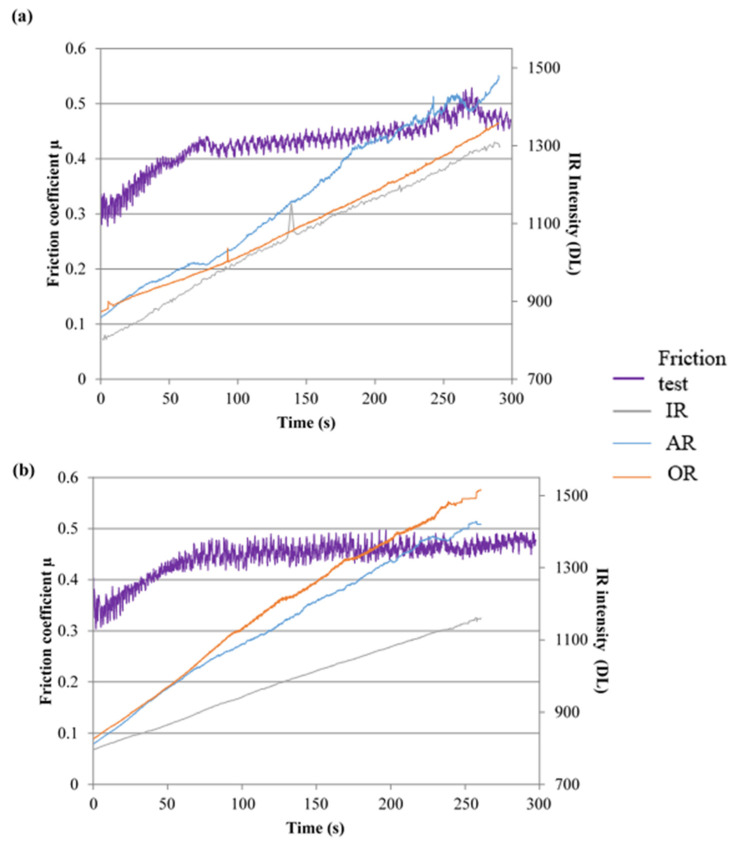
Friction coefficient evolution correlated with Infrared Radiation (IR) evolution over time for (**a**) FMFS and (**b**) FMFBs.

Figure 5 shows that the friction coefficient evolution for FMFS and FMFBs is similar, with a more stable friction coefficient for FMFBs. For FMFS, the increase in friction appears to be disrupted on three occasions for the 5 min test; at 42 s, 73 s, and 262 s, a phase of friction stabilisation appears to follow each event (Figure 5a). For FMFBs, the friction evolves from 0.34 to 0.46 with a transient phase of friction coefficient from the test beginning until 95 s, followed by a plateau reflecting friction stabilisation around 0.46 (Figure 5b). The friction fluctuations are smaller to those observed for the friction test on FMFS (Figure 5). This suggests that the compliance of the rubbing surfaces is established more quickly for FMFBs.

Figure 5 gives the IR radiation evolutions on the OR, AR, and IR of the disc track associated with the friction coefficient evolution in order to correlate the tribological and infrared events. In Figure 5, the comparison of the IR radiation intensity at the IR, AR, and OR with the friction coefficient evolution shows a correlation with the AR and IR of the disc track.

### 3.3. Thermal Phenomena Correlation

The results of infrared thermography display the thermal radiation intensity of the disc-rubbed surface overlapped with the pin surface image after the sliding test.

The scene observed in infrared corresponds to the disc surface coming out of contact with the pin. Figure 5 gives the IR radiation evolutions on the OR, AR, and IR of the disc track, associated with the friction coefficient evolution. For FMFS, during the intervals 47 s–79 s and 233 s–282 s, the rise in friction coefficient coincides with the rise in IR intensity on the AR (Figure 5a). At 243 s and 261 s, two abrupt variations of emissivity, observed on the AR, corresponding probably to the release of third-body particles in the contact, were recirculated by the disc. These two events, which imply a local reorganisation of the third body in the contact, coincide with a punctual rise in the friction coefficient for FMFS. Similarly, at 139 s, the IR peak observed on the IR corresponds to a circulation of a larger deposit of a third body. A high peak in the IR intensity at 167 s at the AR corresponds to a rather abrupt decrease in the friction coefficient (Figure 5a). This change of emissivity indicates a third-body emission recirculated on the AR of the disc.

In Figure 5b, a comparison of the IR intensity at the RI, RM, and RE radii with the evolution of the friction test shows a correlation of fluctuations in friction coefficient and IR radiation at the RM and RI radii. No sudden thermal events were observed for FMFBs contrary to FMFS. The higher IR radiation at these two radii indicates a more important thermal localisation in this area of the friction track.

**Figure 6:**
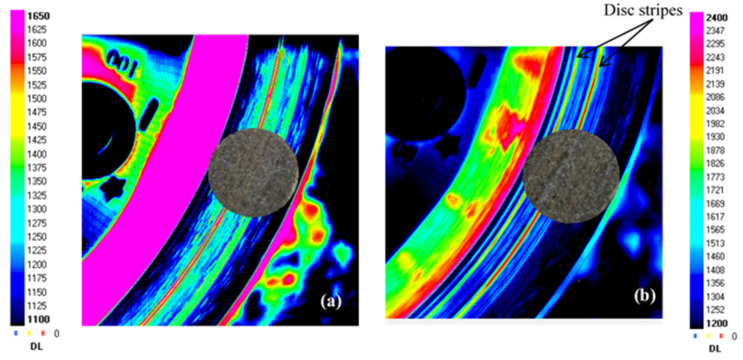
Infrared radiation of the same area of the disc rubbing against (**a**) FMFS and (**b**) FMFBs at the end of 5 min friction test.

The thermograms in Figure 6a show that the contact is fairly broadly centred on the AR for FMFS at the end of the friction test. Figure 6b indicates that the contact is more localised at the IR and towards the AR at the end of the friction test for FMFBs. The presence of two stripes on the disc is also identified using these thermograms (Figure 6b). Indeed, a groove offers a larger IR radiation surface than a flat surface, which corresponds to a very high apparent emissivity that makes it visible in infrared.

It is generally accepted that the thermal localisation phenomena involved in friction correspond to the localisation of load-bearing zones. These phenomena interact with the tribological circuit through the activation of source flows and recirculation flows [25]. Indeed, friction variations are revealing of changes at the interface in the load accommodation mechanisms, sliding speed, and energy dissipation, which result from the evolution of the third-body layer and related material flows [26,27].

### 3.4. Friction and Wear Mechanisms

#### 3.4.1. Wear Mechanisms of FMFS

**Figure 7:**
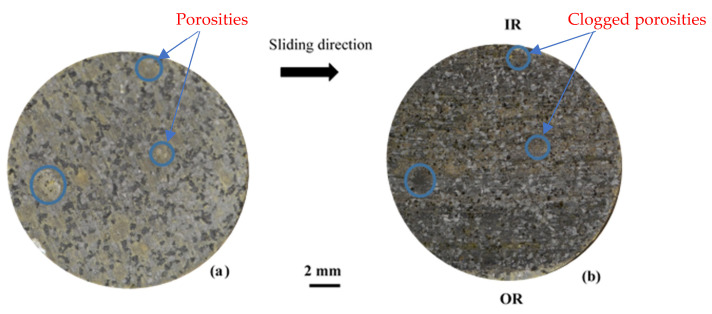
Photos taken before (**a**,**b**) after the friction for FMFS.

The damage and wear mechanisms induced by friction were investigated by means of Scanning Electron Microscopy (SEM) observations and correlated with infrared thermography results. Photos taken before and after the friction tests provide a macroscopic visualisation of the entire pin surface (Figure 7). Porosities induced by fibre entanglements, circled in blue in Figure 7, are completely obstructed by the third-body debris at the end of the friction test for FMFS.

A nonhomogeneous distribution of the third-body and the load-bearing plateaus, reflected by several zones of the rubbed surface with different colours, is noticed. SEM observations were performed on three zones: the outer (OR), average (AR), and inner (IR) radii of disc track. Observations are considered in relation to the location of the contact observed at the end of the friction test, on the average radius (AR), and towards the outside (OR) of the friction track.

**Figure 8:**
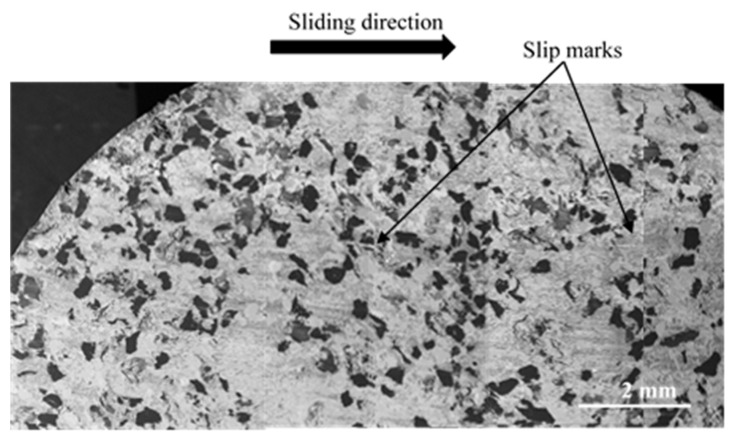
Distribution of powdered third-body and flat plates at IR and AR for FMFS (SEM and BSE).

In the following, for all micrographs, the sliding direction is indicated by an arrow. The observation of the inner (IR) and average (AR) radii indicates a heterogeneous distribution of powdered third-body and flat plates for FMFS (Figure 8). Indeed, the dark grey areas correspond to the flat plates of the third body and those in light grey to the powder beds. Slip marks are visible on some of these plates. 

**Figure 9:**
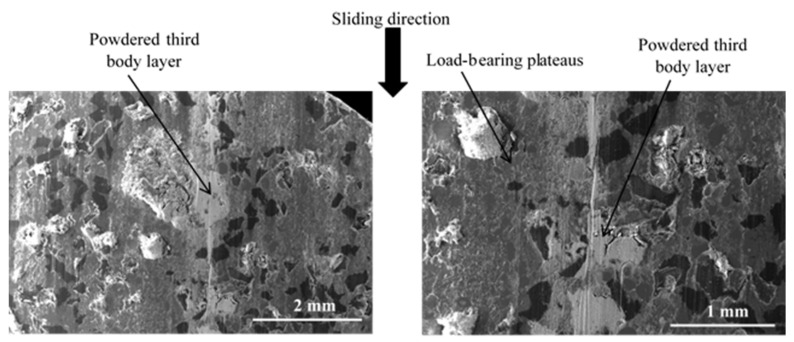
Open contact area at the AR for FMFS (SEM and BSE).

At the AR (Figure 9), SEM observations show the presence of a powdered third-body layer (light grey) that correspond to an open contact. These powdered layers are extended in the sliding direction and alternate in the radial direction with load-bearing plateaus (dark grey).

**Figure 10:**
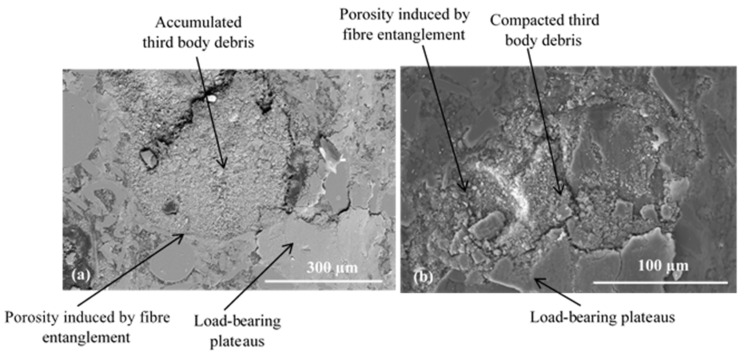
Load-bearing zone at AR for FMFS (SEM, (**a**) BSE and (**b**) SE).

**Figure 11:**
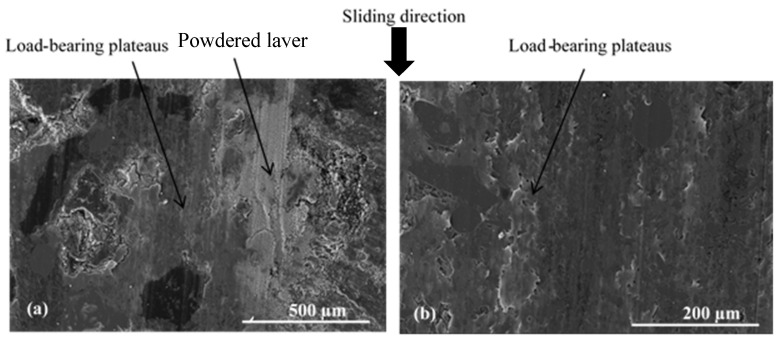
Open contact area and load-bearing zone at the OR for FMFS (SEM, mode SE) showing (**a**) powdered layer and (**b**) extended load-bearing plateaus.

For FMFS, the area extending from the AR to the OR corresponds to the contact location according to the thermographic analysis. Figure 10 shows the presence of load-bearing flat plates that are developed over a significant part of this contact area. In Figure 10a,b, the voids induced by the fibre entanglements and rubber are surrounded by flat plates. The porosities are clogged with compacted third-body particles. Thus, this contact area location is rich in flat plates, which correspond to the load-bearing zone. Some powdered layers and many load-bearing plateaus are developed at the OR, which include an open contact area and load-bearing zone (Figure 11).

Porosities induced by fibre entanglements, located on the AR, are covered with dense and accumulated third-body particles (Figure 10a,b). These particle accumulations trapped in the porosities are surrounded by load-bearing plateaus. It should be noted that the development of these load-bearing plates, also known as “secondary” contact plates, continues through the accumulation and compaction phase sequences. Thus, the plates expand and can, through coalescence, reach several hundred micrometres in size as shown in Figure 11b. Plates up to 300 µm in size surrounding the porosity clogged with third-body debris are shown in Figure 11b.

From these observations, we can notice that porosities generate an irregular supply of the tribological system, which disturbs load-bearing mechanisms [26]. In addition, large porosities disrupt the developing of the third-body layer and disadvantage load-bearing expansion. These results are in accordance with previous studies that report that the wear mechanism plays a dominating role to characterise interactions between the rubbed surfaces. The presence of the accumulated wear debris and entrapment results in the formation of third-body layers and has an influence on the friction stability and wear rate [23,27,28].

#### 3.4.2. Wear Mechanisms of FMFBs

The following paragraph is a comparison with FMFBs in order to highlight the link between microstructural characteristics and tribological behaviour of the friction material.

**Figure 12:**
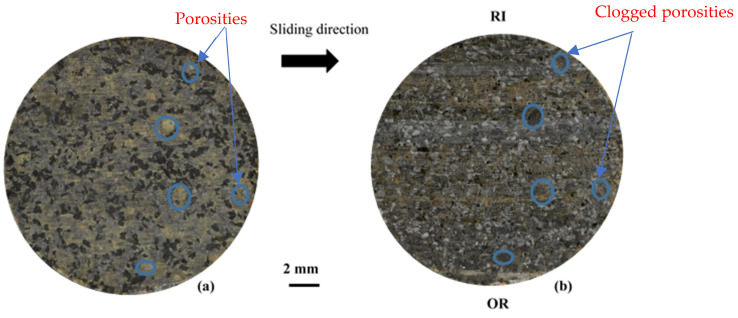
Photos taken before (**a**,**b**) after the friction tests for FMFBs.

On a macroscopic scale (Figure 12), the fibre ball-induced porosities, circled in blue, are completely filled with the third body after the test. Several zones of the rubbed surface at the RI, RM, and RE radii are distinguishable at this scale in relation to the sliding direction. They are the subject of detailed SEM observations.

**Figure 13:**
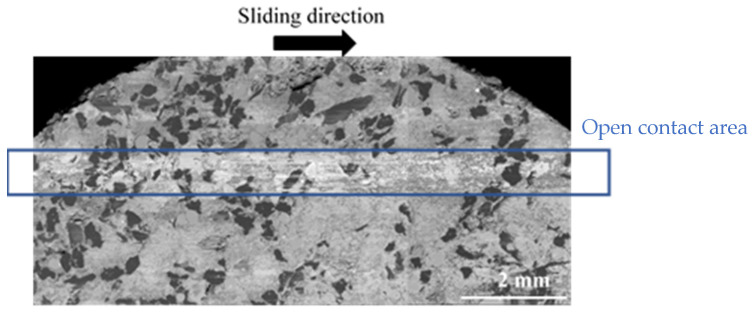
Open contact area at the IR (SEM and BSE).

**Figure 14:**
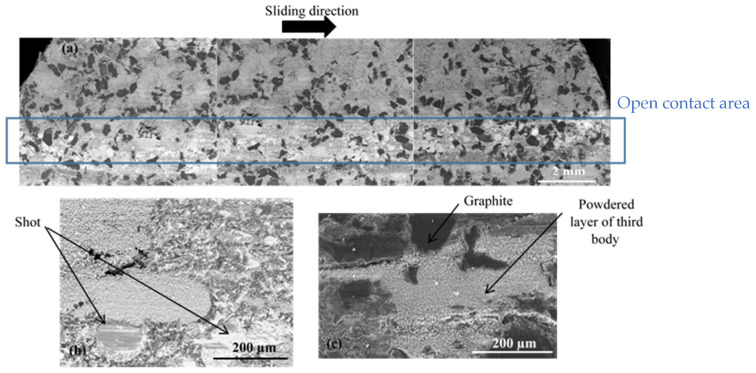
Open contact area at AR (SEM, (**a**,**b**) BSE and (**c**) SE).

The areas corresponding to the stripes (Figure 6b) have an open contact at the IR and towards the AR. They consist of accumulated third-body particles (in light grey), which are free to flow and do not form load-bearing plateaus (Figure 13 and Figure 14).

Figure 14b,c show rockwool shots and graphite that are shaved and held anchored to the matrix. They constitute primary third-body plateaus, as shown by powdered third-body debris on the surface, surrounded by uncompacted powder islands.

**Figure 15:**
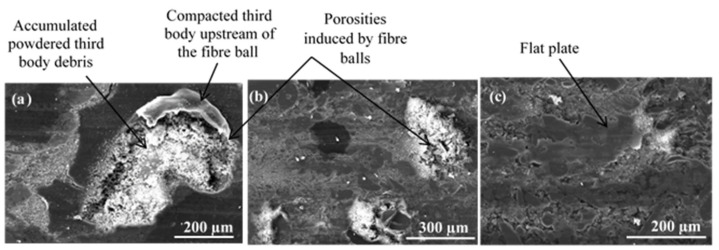
Load-bearing zone at IR (SEM, SE) with (**a**,**b**) porosities induced by fibre balls and (**c**) flat plates.

Porosities induced by fibre balls, located in the load-bearing zones, especially at the IR, are more filled with third bodies and lined with secondary third-body plateaus upstream of the fibre balls (Figure 15).

**Figure 16:**
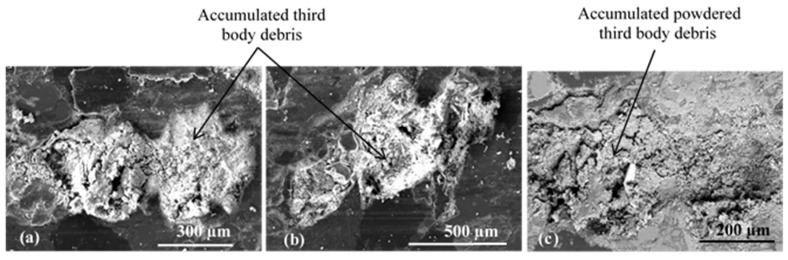
Tribological behaviour of fibre balls located at open contact zone at IR (SEM, (**a**,**b**) SE and (**c**) BSE).

The powdered layers are extended in the direction of sliding. Accumulations upstream of the rockwool fibres are observed, impeding particle flow. Porosities induced by fibre balls, observed at the open contact zone at the IR, contain dense accumulations of uncompacted third bodies (Figure 16). This shows the important contribution of porosities homogeneously distributed over the surface of the material, to the trapping and accumulation of the third body and the development of the load-bearing plateaus in an extensive and uniform manner.

**Figure 17:**
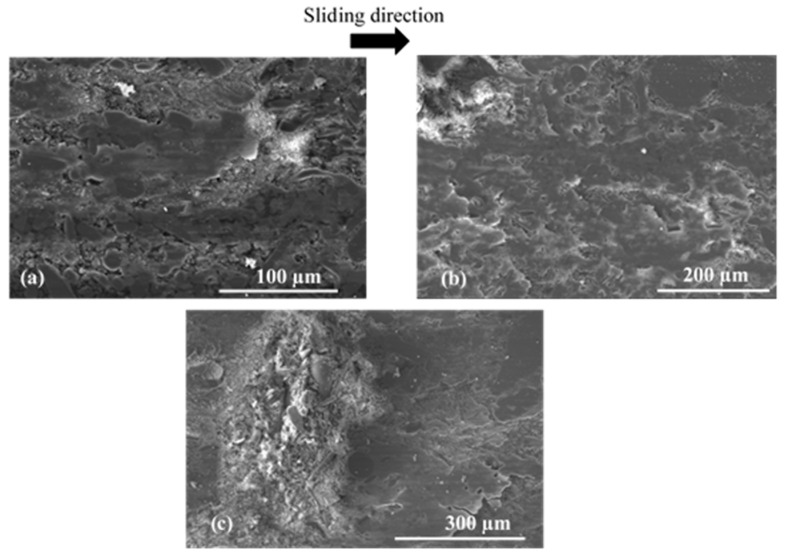
Secondary third-body plateaus located at the load-bearing zone at (**a**) IR, (**b**) AR, and (**c**) OR.

Numerous extensive secondary third-body plateaus are present in the load-bearing zones at AR and IR and also at OR with less of a contact location (Figure 17). The high number of flat load-bearing plateaus and their extent in the different zones of the rubbed surface explain the shape of the friction transient phase, which reaches faster stabilisation than FMFS. Indeed, the friction maintains better stability when a regular third-body layer is established between the two contact surfaces [29,30].

These observations show that for FMFBs, the production of third-body particles is greater and more extensive on different areas of the rubbing surface than that of FMFS. Porosities of FMFBs, homogeneously distributed in the contact, favour the development, coalescence, and maintenance of load-bearing plateaus in the contact thus ensuring more stable friction. Thus, wear mechanism analysis confirms that the constituent distribution of FMFBs is more prone to form regular and stable third-body layers, which further explain its better tribological behaviour than FMFS. This result is in accordance with several studies showing that the homogeneous distribution of secondary plateaus over the rubbed material, such as in FMFBs, generates a more homogeneous transfer film on the counterface, which induces better friction performance [3,31]. Liu et al. [32] also demonstrated that the continuous transfer film might act as a lubricant layer, which leads to a decrease in the friction coefficient and the wear rate of rubbing surfaces of the pin-on-disc system.

### 3.5. Wear Behaviour

Table 2 presents the temporal mass temperature variations measured in the disc and the pin during friction tests for FMFS and FMFB materials. The results of the temporal mass temperature of the pins of the two materials are comparable. However, the disc heats up more when rubbed against FMFBs compared to FMFS. In this case, the surface temperature variation is driven by the thermal contact resistance, which depends in particular on the third body at the interface and not on a difference in thermal effusivity of the materials, since their values are close [16]. This observation is consistent with the analyses of rubbed surfaces of the pins. Regarding the FMFB material, its higher surface porosity, due to fibre balls, compared to FMFS could act as a thermal insulator and consequently increase the thermal resistance of the friction interface reducing the effective contact area, thus limiting the heat transfer [33].

The wear rate was determined for the two materials by measuring the thickness of the pin before and after the friction test. It is revealed that the specific wear is higher for FMFS with 5.7 µm/km compared to 2.7 µm/km for FMFBs. This could be explained by the presence of more numerous secondary third-body plateaus in FMFBs that protect the rubbed material surface [30,34].

Porosities induced by fibre entanglements in FMFS, presenting irregular distribution and large size on the friction surface, disturb the stability of friction and involve low wear resistance. In fact, they generate an irregular supply of the tribological system, which disturbs load-bearing mechanisms [26]. In addition, large porosities disrupt the development of the third-body layer and disadvantage load-bearing expansion. This result is consistent with several previous studies that indicated that when load-bearing plateaus are homogeneously distributed over the rubbed material surface, as in FMFBs, better friction performance is achieved [3,31]. Elzayady and Elsoeudy [35] also showed that the third-body film reduces the surface harshness and thus enhances the strength of the material and minimizes the wear rate. In addition, Bernard and Jayakumari [36] indicated that the dimensions and composition of load-bearing plateaus have a great impact on the frictional stability of brake lining materials.

## 4. Conclusions

This work established the link between microstructural heterogeneity induced by the rockwool fibre arrangement and tribological behaviour of friction materials. The comparison of the friction and wear behaviour of simplified formulations, which are distinguished by their heterogeneity, leads to several conclusions:

Friction appears to be a little dependent on the microstructural heterogeneity of the two simplified formulations. It was demonstrated that:

- A high source flow provided by homogeneous distribution of fibre balls leads to the rapid establishment of a third body in the interface capable of providing bearing capacity and velocity accommodation.
- Locally high surface porosity, due to the fibre entanglements, favours the accumulation of debris of a third body. These particle accumulations, periodically released into the tribological circuit, disrupt the load-bearing plateaus and thus the friction.
- A homogeneously distributed porosity, induced by fibre balls, in the contact favours the development, coalescence, and preservation of the load-bearing plateaus in the contact and thus a stable friction.

Concerning wear behaviour, rockwool fibre arrangement and distribution on the material surface influence the wear mechanisms. It was revealed that:

- Porosity induced by fibre balls and entanglements constitute third-body supplies feeding of the tribological circuit.
- Fibre entanglements lead to a heterogeneous and poorly distributed porosity, which results in an irregular supply of the tribological system.
- Irregular third-body feeding, induced by fibre entanglements, disturbs load-bearing mechanisms and generates a high wear rate.

## Figures and Tables

**Table 1:** Composition and selected particle size of simplified formulations (Friction Material with Fibre Balls (FMFBs) and Friction Material with Separated Fibres (FMFS)).

Classification	Constituents of Simplified Formulation	Weight Proportion (%)	Selected Particle Size (µm)
Binder	Phenolic resin	15.3	<20
Fibres	Rockwool fibres	39.1	Material FMFS: Fiber balls: 400–1000
			Material FMFBs: Fibres < 400
Lubricant	Graphite	11.9	212–300
Abrasive	Alumina	1.2	<20
Friction modifier	Rubber	11.3	325–400
Filler	Calcium carbonate	21.3	<20

**Table 2:** Pin and disc temporal mass temperature variation for FMFS and FMFBs.

	ΔT Disc (°C)	ΔT Pin (°C)
FMFS	20	17.1
FMFBs	22.3	16

## Data Availability

The data presented in this study are available on request from the corresponding author.
